# New Appliances and Things Medical

**Published:** 1895-03-16

**Authors:** 


### NEW APPLIANCES AND THINGS MEDICAL.

[We shall be glad to receive, at our Office, 428, Strand, London, W.C., from the manufacturers, specimens of all new preparations and appliances which may be brought out from time to time.]

### CEREBOS SALT.

(Cerebos Salt Company, Newcastle-on-Tyne.)

The physiological significance of calcium phosphate in being an essential food stuff is apt to be lost sight of in modern dietaries. Whilst numerous firms cater for food stuffs rich in easily assimilable nitrogenous substances and carbo-hydrates in different forms, the cure for rickety children is sometimes forgotten in the modern methods of bread making. Wholemeal bread, although largely advocated, is seldom used, and when one realises that in fine wheat flour the larger proportion of the mineral constituents of the grain are eliminated, it seems desirable that this loss should be supplemented in some way. From our analysis of Cerebos Salt we find that it consists of a mixture of ordinary salt with calcium phosphate in a fine state of division and in a condition which renders it easy of solution in the acid gastric juice. There can be no doubt that in many cases it is necessary to supplement the food supply with phosphates, and the various chemical foods which are largely used for infants and growing children could be dispensed with if Cerebos Salt were used for seasoning. The preparation is well manufactured and is sold at a reasonable price.

